# Diphtheria in a Swiss Asylum Seeker Reception Centre: Outbreak Investigation and Evaluation of Testing and Vaccination Strategies

**DOI:** 10.3389/ijph.2024.1606791

**Published:** 2024-04-24

**Authors:** Lisa Brockhaus, Pascal Urwyler, Ulrike Leutwyler, Eva Würfel, Malte Kohns Vasconcelos, Daniel Goldenberger, Peter Michael Keller, Sarah Tschudin Sutter, Niklaus Daniel Labhardt

**Affiliations:** ^1^ Division of Clinical Epidemiology, Department of Clinical Research, University Hospital of Basel, Basel, Switzerland; ^2^ Department for Infectious Diseases and Hospital Hygiene, University Hospital of Basel, Basel, Switzerland; ^3^ Organisation for Refugee Services, Basel, Switzerland; ^4^ Cantonal Office of Public Health, Basel, Switzerland; ^5^ Department for Infectious Diseases and Vaccinology, University Children’s Hospital Basel, Basel, Switzerland; ^6^ Institute of Medical Biometry and Epidemiology, University Medical Center Hamburg-Eppendorf, Hamburg, Germany; ^7^ Division of Clinical Bacteriology and Mycology, University Hospital of Basel, Basel, Switzerland

**Keywords:** diphtheria, corynebacterium diphtheriae, outbreak, migrant, asylum seeker

## Abstract

**Objectives:** To describe a suspected diphtheria outbreak in a Swiss asylum seeker reception centre, and to analyse its management response regarding testing and vaccination.

**Methods:** We retrospectively analysed clinical, microbiology, and case management data of all asylum seekers tested for *C. diphtheriae* between 28th August and 31st December 2022 while residing at the centre. Results are reported descriptively.

**Results:** Among 265 individuals tested, ten cases of cutaneous diphtheria, one simultaneous respiratory and cutaneous case, and nine respiratory carriers were identified. Mass throat screening, targeted throat testing and targeted wound testing yielded 4.8%, 4.3%, and 17.4% positive results, respectively. No respiratory carrier was identified among cutaneous cases undergoing a throat swab, and no symptomatic case was identified among individuals with unspecific throat symptoms. Rates of vaccination implementation of newly arriving asylum seekers before and after the outbreak were low (17.5% and 15.5%, respectively), as were rates of targeted vaccination among cases and close contacts.

**Conclusion:** We provide evidence for transmission both prior to arrival and within the setting, suboptimal practices and timeliness of testing, and implementation gaps in vaccination.

## Introduction

Diphtheria has re-emerged as a significant health concern within refugee settings across Europe, with 318 cases reported through the European Surveillance system in 2022, and numbers in 2023 staying on a similar level [1, 2]. This resurgence of diphtheria prompted substantial clinical and public health efforts to control the disease, given the high mortality in unvaccinated and untreated cases [3]. However, the limited research interest over the last decades resulted in substantial knowledge gaps regarding the epidemiology and control of diphtheria [3]. However, seroprevalence studies among refugee populations and World Health Organisation country estimates suggest that vaccination coverage against diphtheria may be below estimated herd immunity thresholds in some countries currently representing high proportions of asylum seekers in Europe, including Afghanistan and Syria [4–6].

Recommendations for infection prevention and control (IPC) in migrant settings include droplet isolation for confirmed and suspected cases of respiratory diphtheria, contact precautions including dressing of wounds for confirmed and suspected cases of cutaneous diphtheria, testing of close contacts, reinforcement of vaccination, and, in part, contact restriction and antimicrobial post-exposure prophylaxis for close contacts [7–9]. Nevertheless, it has also been suggested that a targeted approach involving contact tracing, testing and prophylaxis may be unsustainable and ineffective in challenging environments such as high-turnover reception centres, leading to the recommendation of mass antibiotic prophylaxis and mass vaccination in England in November 2022 [8].

Similar to other western European countries, Switzerland saw a surge in asylum applications by 64% in 2022, peaking around October as is regularly observed across western Europe [10, 11]. While there are conceptual differences in reception systems between European countries, e.g., regarding intended length of stay before transfer to other accommodation, insufficient reception capacities are a chronic problem across Europe [12, 13]. This shortage was even more pronounced in late 2022 [14]. As described for initial accommodation centres in other countries [12, 13], asylum seeker reception centres in Switzerland are characterised by a high turnover of residents, fluctuating admission rates, and short notice for resident transfers to other facilities. The living conditions typically consist of dormitories and shared amenities, and formal isolation facilities do not exist. Resources of healthcare professionals are limited on site.

This study aims to provide a detailed description of a suspected diphtheria outbreak in a Swiss national asylum seeker reception centre in 2022, and to explore and analyse the implementation of infection control measures which were guided by national ad-hoc recommendations [9]. Additionally, we aim to offer a contextual perspective from staff members on key findings.

## Methods

We retrospectively analysed clinical, microbiological, and case management information of all asylum seekers residing at one national reception centre in Switzerland, who underwent at least one test for *C. diphtheriae*, between 28th August (date of the first case testing positive) and 31st December 2022. We refer to this investigation as an outbreak investigation adopting a generic definition of an outbreak as “the occurrence of more cases than expected in a particular population, in a specific geographical area and over a specified period of time,” a definition suggested for field epidemiology investigations by the European Centre for Disease Prevention and Control [14]. Methods and Results are reported along the STROBE guidelines. Ethical approval was obtained from the Ethics Committee of North-west Switzerland (2023-00771).

### Study Setting

The asylum seeker reception centre at which this study was conducted, accommodates individuals awaiting primary review of their asylum applications before they are transferred to regional (Swiss cantonal) centres. The centre is governed by national authorities and its infrastructure can accommodate approximately 400 residents. The living environment is composed of 12-person dormitories and shared amenities. It includes a floor with 150 beds designated for unaccompanied minors which, during peak periods, regularly exceeds capacity. Apart from a voluntary, questionnaire-based medical admission check, the primary medical point of contact is a nurse-led clinic that has limited referral options.

Ad-hoc guidance for diphtheria infection prevention and control was published on 4th October 2022 by national health authorities, following first cases being reported from various parts of Switzerland [9]. Key recommendations regarding testing were throat-testing of individuals with signs of classic respiratory diphtheria (adherent membrane, barking cough or stridor), of close contacts of any identified case, and of individuals receiving a wound test in the case of high clinical or epidemiological index of suspicion. It further recommended testing of any wound with chronic appearance. Regarding immunisation, proactive offer of catch-up vaccinations to new arrivals, including diphtheria-tetanus-pertussis (DTP), has been recommended since 2018, if vaccination history cannot be established [15]. Furthermore, ad-hoc guidance suggested vaccinating close contacts immediately, and cases after convalescence, if previous vaccination was not documented. Implementation of the measures was primarily in the responsibility of the reception centres, including transfer of the necessary information in the case of transfer of individuals to other accommodations.

### Data Collection

Clinical and case management information of this population was retrieved from paper-based medical records of the centre (as documented by nursing professionals employed at the centre) and supplemented by staff input when necessary. Electronic medical records of University Hospital Basel and University Children’s Hospital Basel were consulted when applicable. This included basic demographic data, reasons for testing, clinical course if tested positive, decisions regarding antibiotic treatment, isolation/quarantine, antimicrobial chemoprophylaxis, diphtheria-tetanus-pertussis (DTP) vaccination, and hospital referrals.

Documentation practices were discussed in depth with the nurses at the centre to ensure to limit our analysis to valid variables. Data on chemoprophylaxis, antibiotic therapy, and quarantaine and isolation practices were thus excluded.

Microbiological data including sample types, analyses, results, and date of sampling was obtained from the University Hospital Basel microbiology laboratory database, irrespective of the sender (including samples collected during hospital consultations). This was the sole receiving laboratory for samples from asylum centres in the region to be tested specifically for *C. diphtheriae*. Multiple samples of the same individual from different testing dates were included, while multiple wound samples from the same testing date, and follow-up samples in positive individuals for clearance confirmation, were excluded.

Individual-level administrative data, such as arrival and departure dates, provisional status as an unaccompanied minor asylum seeker (UMA), and recorded age at departure, were retrieved from the Swiss national asylum seeker database.

Linkage between data sources was accomplished using the Swiss national asylum seeker number as a unique identifier.

### Outbreak Description

We allocated individuals with a positive test using the following case definitions [8]: (i) confirmed respiratory case (upper respiratory tract infection (URTI) symptoms consistent with classic respiratory diphtheria (adherent membrane) or other symptomatic presentation including mild URTI symptoms, with laboratory confirmation of toxigenic *C. diphtheriae*); (ii) confirmed cutaneous case (cutaneous lesion(s) of any appearance with laboratory confirmation of toxigenic *C. diphtheriae*), (iii) asymptomatic respiratory carrier (laboratory confirmation of toxigenic *C. diphtheriae* from a throat swab, in the absence of any clinical symptoms).

Potential transmission within the setting was assessed by comparing pre-test durations of stay with modelled estimates of incubation periods for respiratory diphtheria, and throat colonisation time in untreated individuals, from relevant literature sources [3].

### Assessment of Infection Control Implementation

#### Testing

Each individual test was categorised based on the indications for testing as suggested by the national ad-hoc guidance [9].

#### Vaccination

DTP vaccination data was analysed for all individuals with available medical records at the centre. Vaccination rates prior to the outbreak were calculated using the population of the primarily affected floor. Vaccination rates prior to the mass throat screening/mass vaccination were calculated using the affected floor’s population. Vaccination rates after the mass throat screening/mass vaccination were calculated using a sample of asylum seekers who arrived after the mass vaccination date (i.e., eligible to regular catch-up vaccination).

### Data Linkage and Statistical Analyses

All data were entered into a REDcap database that served to link data from different sources to the individual. Data were analysed using R, Version 4.2.2. Results are reported descriptively using proportions, medians and interquartile ranges, and confidence intervals where appropriate. Missing data were excluded from the denominator for each analysis separately.

### Key Informant Interviews

Each finding regarding testing and vaccination measures was systematically discussed with two members of nursing head staff employed at the centre (one head nurse, one nursing staff team leader). Questions focused on documentation practices, perceived barriers to implementation, and perceived acceptance of measures among the residents.

## Results

### Study Population

We identified 265 asylum seekers who received at least one test during the observation period while residing at the centre. Basic demographic data were available for 238 individuals, dates of stay for 245, and medical records from the centre were retrieved for 193 (i.e., for 72.8% of the population of interest).

The 265 individuals received a total of 306 tests, of which 264 (86.3%) were performed at the reception centre, and the remainder during hospital consultations.

### Outbreak Description

The first cutaneous diphtheria case was identified on 28th August 2023 a floor dedicated to UMAs who had stayed at the center for 7 days before diagnosis. Testing was prompted by a travel history with an individual testing positive at a different reception centre. A mass throat screening of the respective floor, conducted 4 days after the index case’s notification, revealed a cluster of 7 (4.9%) respiratory carriers.

**FIGURE 1:**
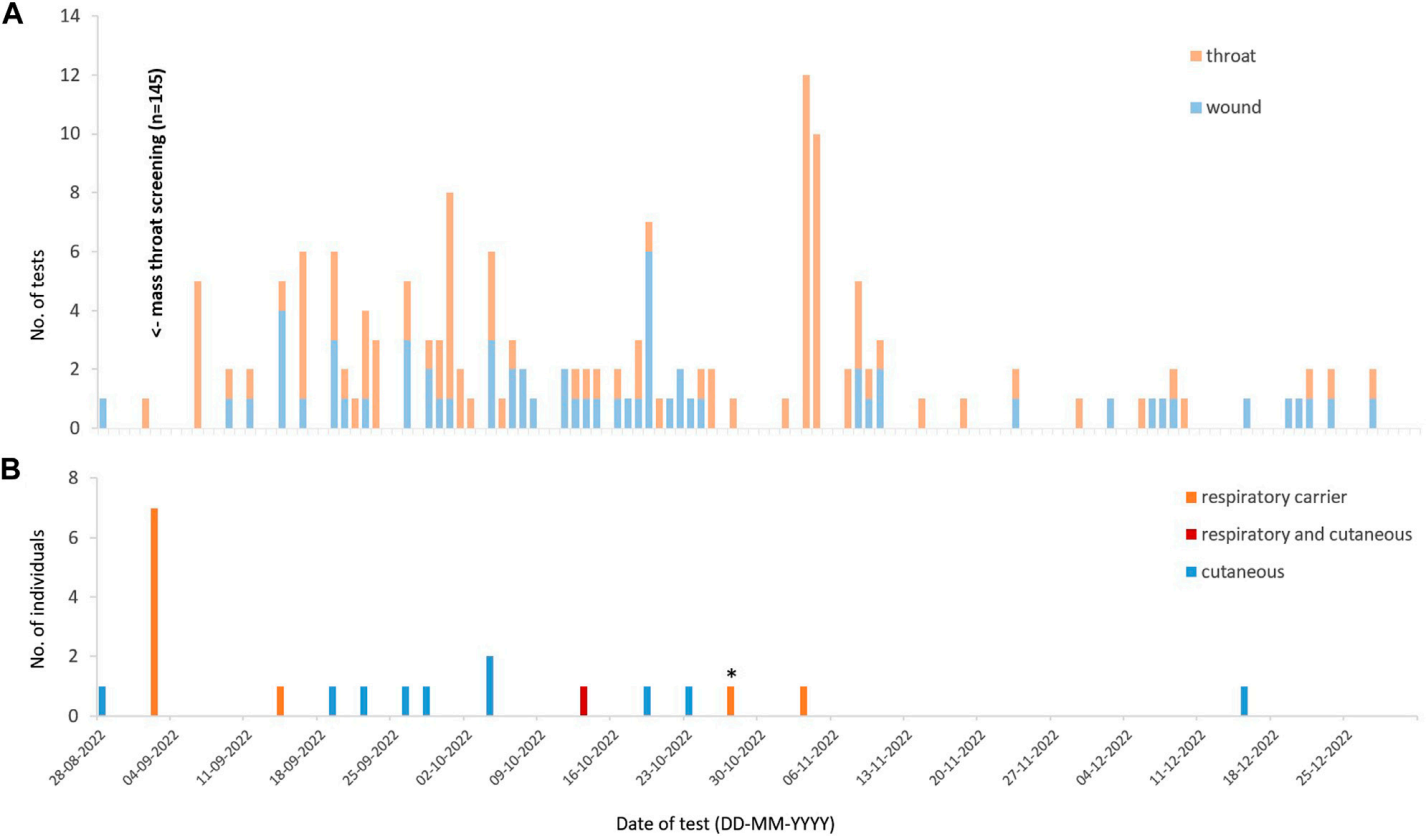
**(A)** Total throat and wound tests performed over the study observation period, by day. **(B)** Individuals testing positive, by day (Switzerland, 2022) *This individual tested positive previously on 02-09-2022.

By 31st December 2022, a total of 20 individuals tested positive: One case of simultaneous respiratory and cutaneous diphtheria, ten cutaneous cases, and nine respiratory carriers. Hence, among the symptomatic cases, 10/11 (90.9%) were cutaneous. Among the individuals with a positive throat test, 9/10 (90.0%) were asymptomatic carriers. Figure 1 illustrates the distribution of positive individuals over time, notably without a peak in symptomatic infections. Among the respiratory carriers, one tested positive on a second occasion despite negative clearance tests in-between. All *C. diphtheriae* isolates tested positive for diphtheria toxin using PCR.

One respiratory carrier had received one dose of DTP vaccination at the centre 7 days before testing positive. The remaining had no documentation of previous vaccination.

All individuals testing positive were male, median age was 17 years (IQR 16.0–18.8, range 14–34), and 18/20 (90.0%) were provisional UMAs from Afghanistan. Two of the 20 individuals did not reside on the primarily affected floor and originated from Iran and Syria, respectively.

### Setting Characteristics

At the time of notification on the index case, the resident population (*n* = 145) of the affected floor was characterised by a median age of 16 years (IQR 16.0–16.4, range 13–24), 97.2% were male, and 87.4% were of Afghan nationality. Their total duration of stay was at a median of 102.0 days (IQR 89.8–120.5; range 9–218). Longitudinal occupancy rates over the whole observation period could not be determined (based on staff interviews, occupancy was generally >90%, with frequent episodes of overcrowding).

### Pre-Test Duration of Stay at the Centre

**FIGURE 2:**
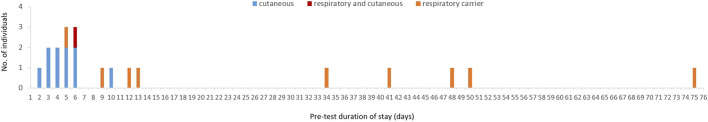
Pre-test duration of stay at the asylum seeker reception centre, of all individuals testing positive (Switzerland, 2022) NB: For the simultaneous respiratory and cutaneous case, the duration of stay before the throat test is indicated.

11 of the 20 individuals testing positive had arrived after the date of the first case being identified. Pre-test durations of stay at the centre were as follows (Figure 2): median 34.0 days (IQR 12.0–48.0, range 5–75) among all respiratory carriers, 34.0 days (IQR 12.5–44.5, range 9–50) in the initial cluster of 7 respiratory carriers; 6 days in the one respiratory case (wound test on day 4, throat test on day 6 following onset of throat symptoms), and 4.5 days (IQR 3.3–5.8, range 2–10) in the cutaneous cases.

Using a pre-test duration of stay of 48 days as a cut-off for a 95% likelihood of post-arrival colonisation [3], we identified 2 of the 9 respiratory carriers who met this criterion. The symptomatic respiratory case had a pre-test duration of stay of 6 days (potentially 5 days before onset of prodromal symptoms), not allowing to rule out post-arrival acquisition of infection considering the estimated median incubation period of 1.4 (95%CI 1.0–1.9) days [3]. Published reference data on colonisation durations of wounds are not available to our knowledge.

### Infection Control Implementation

#### Immediate Outbreak Management

Following notification on the index case (day 0), a mass throat screening (*n* = 145) of residents of the affected floor was implemented (day 4), followed by administration of chemoprophylaxis (Azithromycin 500 mg once daily for 3 days; day 4) for the same population, and isolation of the identified seven respiratory carriers in a separate building (day 7).

Vaccination efforts targeted at the same population began on day 4 (*n* = 32) and were followed by a mass vaccination on day 9 (*n* = 223 including individuals from other floors), with vaccination being mandatory for residents of the affected floor.

#### Testing

After the initial mass throat screening, a targeted case-finding approach was implemented.

Table 1 shows indications for testing, and associated positivity rates.

**TABLE 1:** Positivity rates of diphtheria tests by indication for testing, among residents of a national asylum seeker reception centre (Switzerland, 2022).

Indication for testing	Positive tests (%)	Total tests
**Mass throat screening**	**7 (4.8%)**	**145**
**Case finding approach throat**		
- Throat testing of close contacts of any identified case	1 (2.4%)	42
- Throat testing if presenting with URTI symptoms^a^	1 (5.0%)	20
- Concomitant throat testing if tested for wound infection	1 (3.4%)	29
- Unknown^b^	1 (14.3%)	7
**Case finding approach throat: total**	**4 (4.3%)**	**94**
**Case finding approach wounds** ^c^	**11 (17.4%)**	**63**
**Total tests**	**22 (7.2%)**	**306**

Bold values indicate totals of the respective approach.

^a^ Includes the one simultaneous “classic” respiratory and cutaneous case.

^b^ Includes the case testing positive on a second occasion after negative clearance tests.

^c^ Includes the index case, and the simultaneous “classic” respiratory and cutaneous case.

URTI, upper respiratory tract infection.

Targeted throat testing showed an overall low yield (4.3%). Throat testing in individuals with URTI symptoms identified the one case with simultaneous respiratory and cutaneous diphtheria, with classic clinical presentation. The 19 individuals testing negative presented with unspecific URTI symptoms. Concomitant throat screening in individuals without URTI symptoms but wounds was performed in 29 of 63 individuals and included 7 of the 10 cutaneous cases). One respiratory carrier was identified in an individual with a negative wound test, none, however, among the cutaneous cases.

Case finding through wound swabs showed a higher proportion of positive tests (17.4%). Among wound-tested individuals arriving after notification of the index case (*n* = 43), wound testing was performed at a median of 5.0 days (IQR 4.0–13.0, range 1–90) after arrival.

#### Vaccination

Table 2 summarises implementation rates for DTP vaccination other than within the mass vaccination, including all residents with available medical records lacking documentation of previous vaccination. Of note, vaccination documentation prior to arrival at the centre was not available from any of the 193 records.

**TABLE 2:** Implementation rates of diphtheria-tetanus-pertussis catch-up vaccination among residents of a national asylum seeker reception centre, by observation period or indication for vaccination (Switzerland, 2022).

Time period, or indication for vaccination	No. vaccinated (%)	Total eligible population with available records
−Population		
Before the outbreak	23 (17.5%)	126
−All UMAs undergoing the mass throat screening		
After the outbreak	7 (15.5%; 95%CI 4.9%–26.1%)	45
−All AS arriving after mass vaccination who received at least one test during the observation period^a^		
After convalescence	1 (9.1%)	11
−All AS testing positive who had not received vaccination since arrival		
Post-exposure	2 (14.3%)	14
−Close contacts identified after the mass vaccination and who had not received vaccination since arrival		

^a^ Note this is a sample of the AS population arriving after the mass vaccination.

AS, asylum seeker; UMA, unaccompanied minor asylum seeker.

Implementation of regular catch-up vaccination before the mass vaccination was at 17.5%. Notably, 6/126 (4.8%) of the same population had declined to receive catch-up vaccinations upon medical admission check. Vaccination in the period after the mass vaccination was at a similar level (15.5%). Vaccination as a management component for cases after convalescence, and post-exposure in close contacts, was implemented only in a small minority of individuals.

Those 23 individuals who received vaccination before the outbreak did so at a median of 5.0 days (IQR 4.0, 9.0, range 0–59) after arrival. For individuals arriving after the mass vaccination, vaccination took place at a median of 14.0 days (IQR 13.5–37.0, range 4–78) post arrival.

#### Key Informant Interviews

From a staff perspective, testing measures were progressively well implemented after initial challenges when guidance was not yet established. Testing was well accepted among residents. Testing of close contacts was pragmatically implemented by testing individuals living in the same dormitory. However, it was also highlighted that residents used to autonomously change bedrooms, thereby limiting the chances to identify close contact persons. URTI symptoms were a frequent reason for presentation among the centre population, and symptom-based throat testing was performed by the nurses according to judgement. Concomitant throat screening in individuals without URTI symptoms but wounds was mainly implemented in the case of doubt about additional throat symptoms. Wound testing was performed on any witnessed wound with exudate.

The low routine vaccination rates at the centre were primarily explained by staff through the need for an extra appointment for vaccinations. While the voluntary medical admission check is perceived to be attended by the large majority of residents, the extra appointment for vaccination is rarely being attended. An additional barrier emphasised was the regulatory requirement to vaccinate individuals below 16 years of age strictly within pediatric clinics, that was deemed disproportionate by staff in the light of other pragmatic decisions necessary regarding care of UMAs with no guardian available. Furthermore, costs for an external service provider of the mass vaccination implied prior approval from administrative authorities, thereby contributing to the delay in implementation. Acceptance of the mass vaccination among residents was perceived to be high, notably with individuals declining general catch-up vaccination upon arrival also willing to get vaccinated with appropriate explanations being given.

## Discussion

### Main Findings

In this outbreak, among individuals with a positive throat test, we demonstrate a high proportion of asymptomatic respiratory carriers (90.0%). Among the total of symptomatic individuals, we find a predominance of cutaneous cases (90.9%). Concomitant throat screening in cutaneous cases without URTI symptoms did not identify any case with additional respiratory carriage. Among the respiratory carriers, we identified 2 (20.0%) individuals with a high likelihood of acquisition after arrival.

When analysing tests by indication, targeted testing of chronic wound showed a positivity rate substantially higher (17.4%) than targeted throat testing (4.3%).

Implementation of DTP vaccination before and after the mass vaccination was low (17.5% and 15.5%, respectively) despite low refusal rates (4.8%). Vaccination was rarely included as a management component in close contacts and cases.

### Strengths and Limitations

To our knowledge, this is the first evaluation of the current diphtheria outbreaks in asylum seeker settings in Europe with an additional focus on the real-world implementation of infection prevention and control measures. However, with the data available, effectiveness of the implemented measures is hard to assess.

The cases we describe share personal characteristics, are linked in place and time, and represent more cases than expected in this particular population, as suggested for an field epidemiology outbreak definition [14]. However, given the low number of symptomatic cases, it remains unclear whether the identified cases constitute an outbreak, or are the result of increased testing and higher overall number of asylum seeker arrivals over summer. Thus, our findings regarding testing may be more reflective of a context with some *C. diphtheriae* endemicity in this specific population, rather than of an outbreak situation. Longitudinal turnover and occupancy rates were not available to put the number of identified cases in perspective.

Positivity rates of indications for testing are to be interpreted with caution in our analysis, as they rely on low numbers, and may not reflect different outbreak dynamics. Also, implementation of testing practices may vary between settings, thereby further limiting transferability of our findings. Implementation of vaccination may equally vary between different settings and even institutions. Data on isolation and quarantining practices, and on implementation of chemoprophylaxis, were too fragmentary to be systematically analysed.

Further, our analysis demonstrates a substantial amount of missing data, due to missing medical records including the documentation of vaccination, and failure of data linkage. Notably, in Switzerland, health data of arriving asylum seekers is not available routinely or in an electronic form apart from a medical admission check.

### Findings in the Light of Existing Literature

The high proportion of asymptomatic individuals among respiratory cases corresponds to other current outbreak descriptions in European migrant settings [16–18]. These data contrast a recent systematic review and pooled analysis estimating the proportion of asymptomatic respiratory infections in unvaccinated individuals at 31% (95% CI 18%–55%) [3]. A higher proportion of asymptomatic infections may be interpreted as a sign of prevalent immunisation in this population. However, it may also be due to higher case ascertainment.

A relative predominance of wound cases has equally been reported in other recent outbreaks [16–18]. Besides higher clinical awareness with higher testing, prevalent immunisation may likewise serve as an explanation for this finding. Moreover, the overall prevalence and pathogenesis of chronic wounds in new arrivals may vary with time and population. Notably, prevalence of chronic wounds was reported with 80% of arrivals by small boats in the UK in 2022 [17]. Large proportions of infected skin lesions with detection of toxigenic *C. diphtheriae* have recently been reported to be polymicrobial [18, 19].

As opposed to respiratory carriers, evidence on the duration of colonisation in wounds is not available [3]. From a clinical perspective, considering the time required for wounds to evolve, the relatively short pre-test duration of stay of the wound cases in this analysis provides an argument for acquisition of infection before arrival.

Recent national surveillance data from England demonstrated that individuals with simultaneous positive throat and wound tests were rare (2 out of 73 cases) [16]. This data corresponds to our finding of a low yield of concomitant throat testing of asymptomatic individuals undergoing wound testing, however with low numbers in our investigation. We were not able to identify more literature on combined respiratory-cutaneous cases, or cutaneous cases with throat colonisation.

In our analysis, symptom-based throat testing was performed more deliberately than originally recommended. We did not identify any respiratory case in individuals tested for unspecific URTI symptoms. Notably, other recent publications reporting on outbreaks with larger numbers identified classic symptoms only in a minority of the symptomatic cases [16, 20]. Our analysis does not allow to conclude on the usefulness of testing for unspecific URTI symptoms.

Overrepresentation of Afghan nationals was reported in other recent diphtheria outbreak reports and has been discussed in the context of disrupted immunisation programs [7, 16].

Notably, a seroprevalence survey of asylum seekers in the Netherlands in 2016 showed a diphtheria seroprevalence among Afghan asylum seekers of 65%, thereby the lowest of the migrant nationalities tested [5], and far below herd immunity threshold estimated for refugee camp settings [3].

When criticising low vaccination rates, one must keep in mind that vaccination does not prevent colonisation in the case of *C. diphtheriae*, and its effectiveness for immediate outbreak management is limited by the time needed for development of immunity [3]. However, vaccination was estimated to also decrease transmission by 60% by reducing symptomatic shedding, and to interrupt transmission in outbreaks if R0 <2.3 [3].

Although literature on diphtheria outbreaks and related management in migrant reception settings in Europe is still very limited, reports from Belgium, Germany, and England report operational challenges that were also characteristic of this outbreak: limited isolation facilities, difficult contact tracing due to high mobility of this population, understaffing of reception centres, and fragmented responsibilities [16, 19, 21].

It has been suggested that traditional case finding and contact tracing approaches are not feasible in this setting and need adjustments [19], and that the implementation of a mass programme was more effective than individual-level management as it is suggested by many national guidance documents [16]. While the management we describe after the initial mass testing relied on this approach, our findings of a relevant delay to wound testing, relevant delay to regular catch-up vaccination, and a relevant proportion of missing records, may support this conclusion. Our investigation also adds to the evidence that a relevant proportion of infections happened before arrival at the centre, thereby limiting the effectiveness of interventions aiming to decrease local transmission, as has already been noted in other outbreaks [19, 21].

From an epidemiological perspective, the lack of reliable denominator data in migrant settings has been mourned previously. This would be needed to calculate the number of people eligible for an intervention, or proportion of people covered by an intervention [16], but also for a more robust epidemiological analysis. As mentioned earlier, we were not able to obtain any centre-level occupancy data for the period of this outbreak.

### Conclusion—Implications for Future Research and Policy

We describe a relevant number of diphtheria cases in the population of newly arriving asylum seekers in Switzerland over a 3-month period. Firstly, our investigation provides evidence for both continuous introduction of new cases, but also for transmission within the setting, meaning that both mechanisms need to be addressed with appropriate interventions simultaneously. Secondly, regarding testing, we add to the evidence that concomitant throat testing of asymptomatic individuals undergoing wound testing may be of limited value, and that the relevant count and substantial proportion of positive wound tests indicate a potential need to expedite wound testing for new arrivals. Lastly, regarding vaccination, we demonstrate an implementation gap in catch-up DTP vaccination rates despite substantial efforts being made, however limited to the experience of one large national reception centreWe therefore suggest that in Switzerland early routine vaccination of newly arrived asylum seekers may benefit from strengthening with appropriate interventions.

Sharing this management experience may further contribute to reviewing current diphtheria infection prevention and control guidance with regard to testing indications, setting-specific feasibility, and the resources needed for effective implementation, in Switzerland and in potentially similar contexts.
